# “Water Association” Band in Saccharide Amorphous Matrices: Role of Residual Water on Bioprotection

**DOI:** 10.3390/ijms22052496

**Published:** 2021-03-02

**Authors:** Sergio Giuffrida, Antonio Cupane, Grazia Cottone

**Affiliations:** Dipartimento di Fisica e Chimica Emilio Segrè, Università di Palermo, Viale delle Scienze 17-18, I-90128 Palermo, Italy; antonio.cupane@unipa.it

**Keywords:** biopreservation, trehalose, water, myoglobin, infrared spectroscopy

## Abstract

Saccharides protect biostructures against adverse environmental conditions mainly by preventing large scale motions leading to unfolding. The efficiency of this molecular mechanism, which is higher in trehalose with respect to other sugars, strongly depends on hydration and sugar/protein ratio. Here we report an Infrared Spectroscopy study on dry amorphous matrices of the disaccharides trehalose, maltose, sucrose and lactose, and the trisaccharide raffinose. Samples with and without embedded protein (Myoglobin) are investigated at different sugar/protein ratios, and compared. To inspect matrix properties we analyse the Water Association Band (WAB), and carefully decompose it into sub-bands, since their relative population has been shown to effectively probe water structure and dynamics in different matrices. In this work the analysis is extended to investigate the structure of protein-sugar-water samples, for the first time. Results show that several classes of water molecules can be identified in the protein and sugar environment and that their relative population is dependent on the type of sugar and, most important, on the sugar/protein ratio. This gives relevant information on how the molecular interplay between residual waters, sugar and protein molecules affect the biopreserving properties of saccharides matrices.

## 1. Introduction

It is well known that saccharide glasses protect biomolecules from damages induced by dehydration or high temperatures. Among saccharides, trehalose (α-d-glucopyranosyl-α-d-glucopyranoside), a common sugar in organisms able to survive in anhydrobiosis, has been found to be the most efficient in functional recovery of biomaterials [1,2,3,4]. Strong effort has been devoted in dissecting the molecular basis of the trehalose bioprotective mechanism, resulting in different working hypotheses, as water substitution in dehydration conditions, or an indirect preservation of protein solvation via water entrapment at the protein surface, not to mention the viscosity of sugar matrices, which impairs any loss of secondary and tertiary protein structure [5]. A peculiar mechanism based on its glass transition temperature (T${}_{g}$), higher with respect to its homologues sucrose and maltose [6,7], has been also proposed. In this respect it has been shown that disaccharides can produce homogeneous glasses at any cooling rate, above a definite water/sugar mole ratio [8], which for trehalose, maltose and sucrose is in the same order as that reported for the perturbation of the hydrogen bond (HB) network by sugars [9,10]. However, measurements on other saccharides (dextran, inulin and raffinose) showed that a stable glassy state is not correlated with properties of the HB patterns [11,12,13]. Actually, it has been shown how trehalose efficiency could arise from two factors, a strong hydrogen bonding capability and, at the same time, the ability to produce stable glassy structures in a wide hydration range [8,14,15].

Saccharide amorphous systems containing globular and membrane proteins have been extensively studied at fixed sugar/protein (S/P) ratio with various complementary techniques (for some reviews see in [16,17,18,19,20,21]). A common outcome of many of these studies is that the reduction of protein motions depends on the residual hydration. The protein domain is characterized by a water excess, however trehalose groups may bind the protein polar residues. Trehalose could even form patches around the protein that affect the protein backbone dynamics [22,23], starting to form a protective coating at high sugar/protein ratio [24]. To summarize, trehalose can protect biomolecules by locking their surface either directly or through water molecules bridging the protein to the sugar-water matrix. In this case, the role of water is to mediate a dynamic stabilization [25,26,27,28]. This scenario has been recently confirmed by neutron diffraction experiments combined with empirical potential structural refinement (EPSR) simulations [29,30]. Results from this study shows that trehalose entraps a protective water shell around a small model peptide without direct bonding with it, thus avoiding dehydration induced damages.

Another important factor affecting biopreservation in saccharide amorphous matrices is the S/P ratio. In some reports, present in literature, this value is varied to investigate its effects on the mechanism [31,32,33,34,35,36,37,38,39,40]. In the case of large size proteins, reduction of protein dynamics and thermal stabilization have been reported to not depend on protein concentration in trehalose matrices; conversely, a dependence has been found on sucrose content at high S/P ratios [33,36]. In a study of a monoclonal antibody embedded in freeze-dried matrices of trehalose and sucrose, an optimal concentration has been found stabilizing the sample at room temperature up to 2 years [31]. Calorimetric studies [38] on myoglobin in a trehalose-water matrix show that the stability of the matrix increases with the protein content, as pointed out by an increase of the system T${}_{g}$. On the contrary, the protein thermal denaturation temperature decreases, indicating protein destabilization.

Recently dielectric spectroscopy has been used to investigate the stability of myoglobin in trehalose and sucrose samples at different S/P ratio [40]. With this technique different relaxation process could be detected, namely a β relaxation process corresponding to solvent and protein local fluctuations, and an α relaxation process corresponding to large scale solvent and protein motions. In particular, in samples at very low protein content another β process has been observed in trehalose but not in sucrose, attributed to intramolecular rotations in the disaccharide. This process disappears at high protein concentration. In this case, due to scarcity of water molecules, trehalose molecules are probably forced to form intramolecular HBs hindering the rotation, as it usually happens in sucrose. The stabilizing effect of trehalose below T${}_{g}$ is explained by considering the slowdown of water in these samples, in the presence of trehalose. Above T${}_{g}$, the preserving properties of trehalose are attributed to the coupling of the protein and sugar α relaxation process, which increases at high trehalose concentration.

Another recent study reports analogous dielectric spectroscopy results on samples containing lactate dehydrogenase at various trehalose and poly-lysine concentrations [39]. Also in this case, a relation between the residual water mobility and the solution biopreserving properties is observed, which depends on the trehalose + poly-lysine content. This in particular for the so called γ-relaxations process, which may be related to water molecules tightly bound to protein surface. The intermolecular interactions between poly-lysine and trehalose molecules seems to affect the kinetics of water relaxation process.

The main outcome of all these studies is the key role of residual water in modulating both matrix structure and dynamics, and protein-matrix interactions in saccharide matrices with biopreserving properties. Consequently, it is a worthwhile effort to better characterize the properties of this water, and the HB patterns in which it is involved. In the past, this has been partially accomplished in protein-sugar samples at different hydration with Infrared Spectroscopy (FTIR) experiments [16,33,41,42]. In the case of carbon-monoxy myoglobin (MbCO) the bound CO stretching band (COB, ∼1900–2000 cm${}^{-1}$) was used to study the myoglobin properties, as it is one of its most studied spectroscopic marker [43]. The Water Association band (WAB, ∼2000–2400 cm${}^{-1}$, see Section 3), adjacent to the COB, was exploited to concurrently study the matrix properties. It has been reported how this band can be utilised as an effective spectroscopical probe for the solvent in low-water matrices [42,44], where bulk water signal does not hide the other components. Furthermore, along with the COB, the WAB has already been employed to evaluate the simultaneous thermal evolution of matrix and biomolecule [16,45] in systems at fixed S/P ratio. These WAB analyses were mostly restricted to peak shifts and band area variations, which were found sizably dependent on temperature, hydration, type of sugar or even insertion of biostructures, like proteins and liposomes [46].

In this work we report results from room temperature FTIR measurements of the WAB in dry MbCO amorphous matrices at different S/P ratios and very low hydration. The disaccharides trehalose, maltose, sucrose and lactose, and the trisaccharide raffinose are studied. A previous study from our group had investigated these systems from the point of view of the protein. A detailed analysis of the CO band and its sub-bands (the taxonomic A substates [43]) at different S/P ratio is reported in detail in Giuffrida et al. [37], where it is shown how an optimal sugar-protein formulation could be found that guarantees protein preservation at the minimal water quantity.

In the present work we focus on the matrix properties. Doubtless, changing the S/P ratio implies alteration of water-protein, water-sugar and water-water interactions. By general consensus this has strong implications in bioprotection by saccharides, as it has been already pointed out in a wide spectrum of systems, including myoglobin [38,47]. The motivation behind the present study is to put these aspects together in a single comprehensive scheme, in which multiple (low cost/in-house setup) measurements on different systems are brought together and accounted for in a single framework.

With this aim, in the present work we analyze the alteration of the WAB area and shape with the S/P content, also comparing systems with and without the protein. The analysis is deepened, for the first time, by a detailed decomposition of the WAB into sub-bands. This because their relative population has been shown to be a valuable probe for matrix structure and dynamics in different environments [44]. A differential analysis of maximum peaks and populations of the components allows to have a picture of the HBs networks which are established in the systems and, most important, on how these are affected by the system composition. We will show how results are peculiar to each sugar, and contribute in supporting the evidence of the trehalose highest effectiveness.

## 2. Results and Discussion

**Figure 1:**
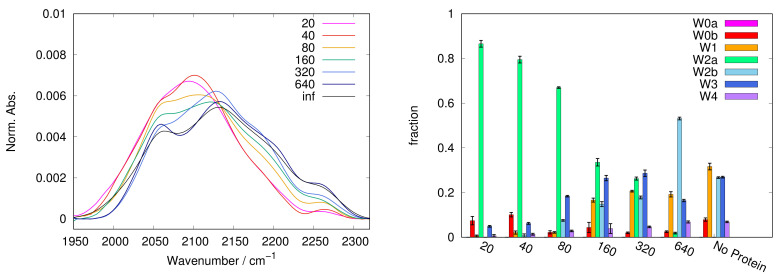
Profile of WAB (**left panel**) in protein + trehalose matrices with various sugar/protein ratios: representative spectra are shown, highlighting the smooth transition from low frequency to high frequency components in the trehalose WAB. Population of the sub-components of the WAB as a function of the sugar:protein ratio (**right panel**); vertical bars are standard deviations on the population. W0a,b are related with water interacting with the protein surface and are present mostly at high protein content. W1 can be attributed to water in chaotropic environment, mostly in the sugar domain. W2a,b correspond to bulk-like water present in the protein (W2a) and sugar domains (W2b), respectively. W3 and W4 correspond to more structured water, and are predominant at high sugar content as well as in sugar-water samples without protein. In particular W4 is assigned to ice-like water. Remarkably only in trehalose this sub-band (2230–2270 cm${}^{-1}$) smoothly populates with the sugar:protein ratio indicating the progressive formation of trehalose patches incorporating water.

**Figure 2:**
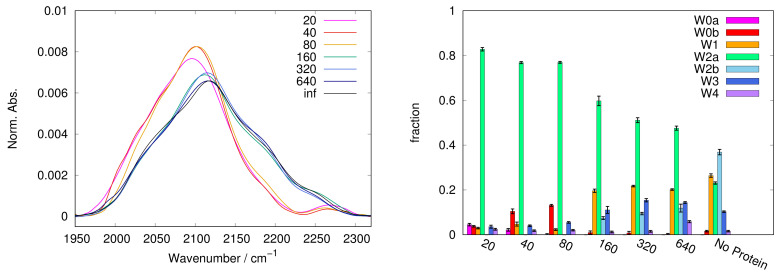
As for Figure 1, in protein + sucrose matrices. At variance with the trehalose case, the transition from high protein (low frequency) to high sugar (high frequency) content is sharp. W2a bulk-like is dominant at almost all sugar/protein ratios.

**Figure 3:**
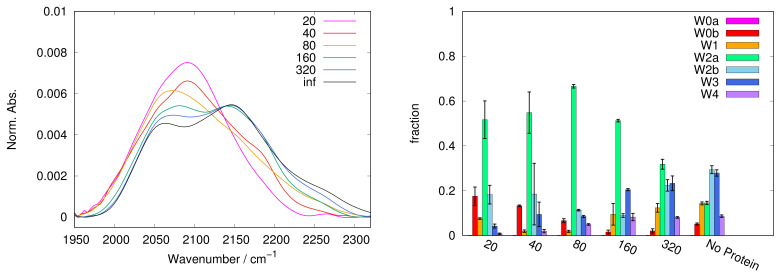
As for Figure 1, in protein + maltose matrices. The WAB behaviour is more similar to the trehalose than to the sucrose case. However, a sizable transition is still observed between S/P = 20 and 40, as well as between 80 and 160.

**Figure 4:**
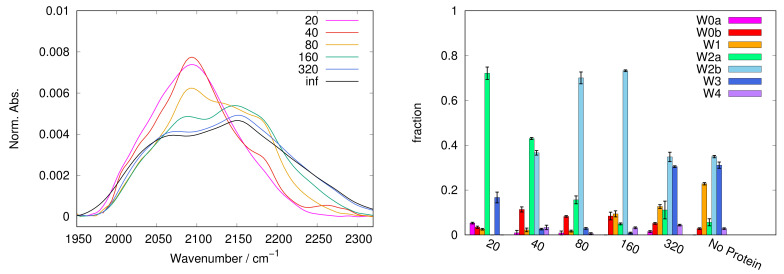
As for Figure 1, in protein + lactose matrices. Here the WAB has a peculiar behaviour with the sugar/protein ratio. The low frequency sub-band in the range 2080–2120 cm${}^{-1}$ (W2a) immediately gives way to the sub-band W2b (see the transition between S/P = 40 and 80), and then progressively to the high frequency components.

**Figure 5:**
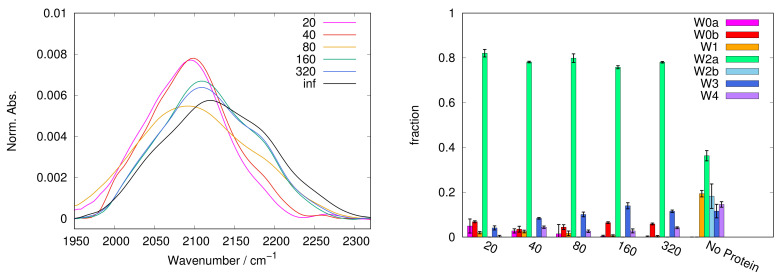
As for Figure 1, in protein + raffinose matrices. Here the WAB behavior looks like the sucrose one. However two distinct steps are evident: between S/P = 40 and 80 and between 80 and 160. In any event, as in the case of sucrose, the W2a bulk like sub-band is dominant at all compositions.

In Figure 1, Figure 2, Figure 3, Figure 4 and Figure 5, left panels, we report the WAB profiles at the various S/P ratios investigated, for each sugar. The profiles show a peculiar behaviour with the S/P ratio, as they form two families. The difference between these families is more pronounced in the case of sucrose, where each group is formed by similar curves. Spectra for systems with low S/P ratio (20 to 80) belong to the first family where low frequency components dominate, resulting in band shapes clearly different from those of protein-free samples, i.e., at infinite S/P ratio (see black lines in Figure 1, Figure 2, Figure 3, Figure 4 and Figure 5). Systems with high S/P ratio (160, 320 and, where present, 640) beget the second family, where high frequency components prevail yielding shapes alike those of protein-free systems. A similar, although less sharp, behaviour was reported for the COB [37], indicating a concurrent alteration of protein and matrix.

Such a behaviour suggests that when the protein and/or the sugar are added, the matrix changes its properties discontinuously. We could identify a transition ratio (S/P = 80), e.g., a threshold between two distinct conditions. Below S/P = 80, water exists in a more chaotropic enviroment [16,44], likely the protein surface. Protein-water interactions lead to a large content of low-frequency sub-bands. Above the threshold, the matrix is largely dominated by the sugar. In this condition, one can conceive that strong sugar-sugar and sugar-water interactions make high frequency components to prevail.

The change between the two regimes can be smooth or abrupt depending on the sugar, and in trehalose it looks remarkably smooth. A peculiar behaviour of trehalose is also evidenced by the results reported in Table 1, where the residual water content after dehydration is reported, expressed as the water/saccharide (W/S) molar ratio. For all saccharides the water content has a minimum at a specific S/P ratio which is almost constant at S/P = 80 (that corresponds to 1:1 myoglobin:sugar mass ratio), irrespective of the sugar. At lower ratios the HB network of the system is dominated by the protein; in this case the sugar cannot provide a complete coating and some more water is required for saturating the HB-forming groups of both the components. In the case of trehalose, where the residual water content at S/P = 40 and S/P = 20 is higher than in the other sugars, this is more evident. According to this molecular picture, above S/P = 80, the protein would become a minority component unable to replace water in the water-sugar network; therefore a certain amount of water is again required to fulfill the HB requirements of the sugar.

In this scenario, at the S/P = 80, myoglobin is able to substitute most completely the water in the sugar-dominated matrix, or vice versa.

From the point of view of the protein, water in the first hydration shell could be indeed replaced by sugar groups, depending on the S/P ratio. In the case of trehalose, this happens at water/sugar mixtures with composition lower than the 2:1 water:sugar stoichiometry. A quantitative picture of the HBs pattern could arise from simulations of proteins embedded in sugar systems, although a comparison with experiments is flawed because simulations are usually carried on a single protein. In a single protein simulation of myoglobin at this water/sugar ratio (i.e., 0.11 g${}_{water}$/g${}_{sugar}$), the estimate of the mean number of different water molecules forming *n* bonds (*n* = 1, …, 4) with the protein was about 90 (to be compared with about 270 at 0.88 g${}_{water}$/g${}_{sugar}$, a dilute sugar solution); the mean number of different sugar OH groups forming *n* bonds with the protein was found about 80 (to be compared with 26 at 0.88 g${}_{water}$/g${}_{sugar}$, which is ten time lower than the number of water-protein bonds) [48]. This is of the order of the S/P threshold above found: at this ratio the sugar could indeed perform a water substitution in the myoglobin domain. At the lower S/P investigated, we found that the residual water content increases; at the lowest ratio (S/P = 20), as reported in Table 1, the W/S value is an order of magnitude higher than the one at S/P = 80. Interestingly, this water excess is of the same magnitude of the ratio of protein-water/protein-trehalose HBs found in the simulation of the diluted sugar solution. Similar results were found in simulations of myoglobin in sucrose or maltose at the same water/sugar ratio [48]. In a multiprotein lysozyme/trehalose/water simulation [49] at similar water/sugar concentrations, it was found that the mean number of trehalose-lysozime and water-lysozime HBs add to about 300, which is almost the estimated mean number of water-lysozyme HBs in aqueous solution. To summarize, at these concentrations, trehalose is able to complement the first hydration shell of similar size globular proteins.

Trehalose samples show a sharp increase on both the sides of the water minimum at S/P = 80, with respect to the other saccharides. This is a hint that trehalose can better absorb and retain water at low hydrations, being able to withstand dehydration until an optimal concentration is reached, where interaction with protein are at maximum (at S/P = 80), and the resulting system is stable even at a very low hydration. This behaviour parallels and helps in rationalizing the smooth variations of the whole WAB profile with S/P. The main outcome is therefore that trehalose matrices promptly adapt to protein and/or co-solute insertion, providing a friendly host environment to the embedded protein. This has strong implications in biopreservation.

**Figure 6:**
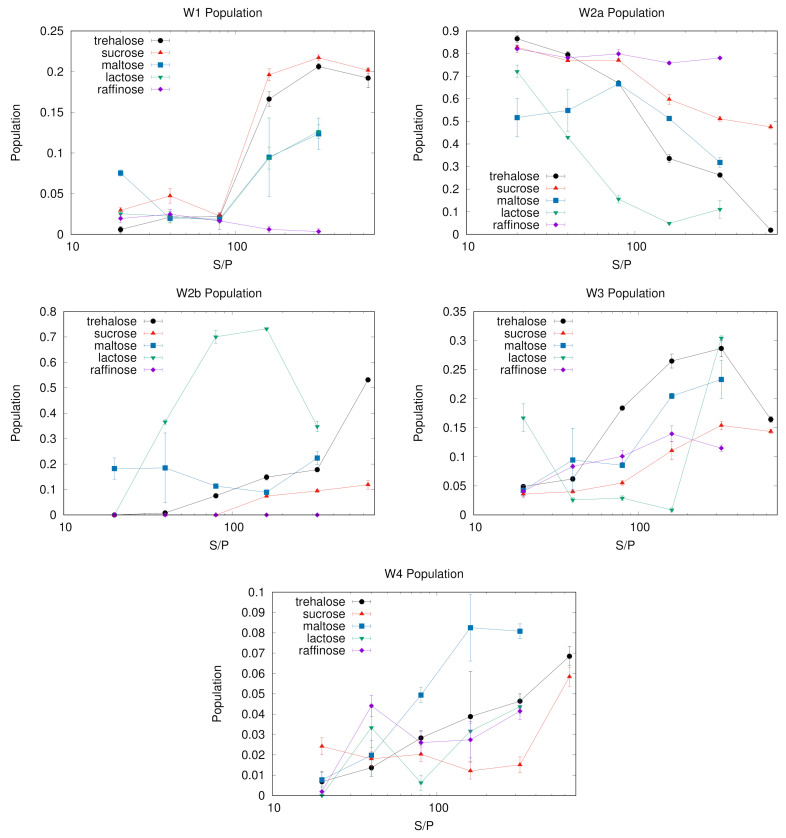
Population of various sub-bands. Vertical bars are standard deviations on the population. W1 is attributed to water in a chaotropic environment, mostly in the sugar domain: its population indeed increases with the sugar:protein ratio, with the exception of raffinose. W2a,b corresponds to bulk-like water present in the protein (W2a) and sugar domains (W2b), respectively: accordingly, W2a decreases with the sugar content while W2b increases, at least in trehalose, maltose and to some extent also in lactose. W3 and W4 correspond to more structured water, and are predominant at high sugar content: their population increase with the sugar:protein ratio, depleting the W2a,b couple.

The dependence of the WAB sub-bands fractional populations (as obtained following the procedure described in Section 3) on S/P ratio are reported in Figure 1, Figure 2, Figure 3, Figure 4 and Figure 5 as histograms (right panels), for the various sugars, and in Figure 6 on a semilogarithmic scale.

As a general observation, data reported confirm that there is a population shift from low frequency to high frequency components with increasing S/P, in almost all matrices, less evident in raffinose. However each sugar exhibits a peculiar behaviour, both with and without protein.

In trehalose the band conversion appears more gradual than in other saccharides. In particular, W2a progressively reduces in favor of W2b (starting from S/P = 80, up to S/P = 640) and of the W3 and W4 sub-bands. In contrast, in sucrose W2a is dominant at almost all S/P values; W2b can be observed only at high S/P, while it becomes dominant in samples not containing protein.

In both trehalose and sucrose, starting from S/P = 160 a gradual increase of W1 is observed. The W1 sub-band, attributed to a chaotropic environment, is present and well populated in almost all samples without protein, while it is scarcely populated at low S/P (<160), where it is consistently less populated that the W0 couple. This behaviour is consistent with a different origin of the two sub-bands, and hints that W0 bands are mainly related to a definitely chaotropic environment, such as the protein surface (but not exclusively as W0b is present also in sugar-only samples), while W1 would be more contributed from water molecules in distorted sugar-water HBs configurations, which therefore prevail by increasing sugar content.

At variance with trehalose and sucrose, in maltose W2b is present already at lower sugar concentrations; however the error bars in these samples are the highest among the sugars investigated in particular at S/P = 20 and 40. We can suggest that this behavior is due to the sugar structure, which is a mixture (roughly 1:1.2 at equilibrium in water) of α and β anomers leading to a larger heterogeneity in the possible protein-sugar-water configurations. This hypothesis is confirmed by the observation that also in lactose, where the two anomeric forms are also present (with ratio 1:1.7 at equilibrium in water), high standard deviations on the populations are found, lower than in the case of maltose, but higher than in the case of the non reducing disaccharides. In maltose W1 remains a minority component, and becomes apparent only at very high sugar concentration (S/P = 320).

In lactose the switch of W2a to W2b starts already at low sugar content (S/P = 40). This behaviour is peculiar of lactose, and deserves an explanation. It is well known that among sugars, and in particular in comparison with trehalose, lactose is the least effective in biopreserving protein [8,50,51], due to a sort of local “phase–separation”, detaching the sugar from the protein. As W2b usually increases with sugar concentration, we could conceive that the W2b population arises from bulk-like water molecules in mainly sugar domains. The W2a to W2b switch observed at low S/P could be then attributed to the set up of lactose domains, “separated” from the protein. The W2b content increases up to at S/P = 160, where it constitutes the dominant component. Then, also in this case, W2b converts in W3. As in the case of maltose, W1 is almost absent at low sugar concentration, while becomes significantly populated in binary matrices as in the case of all other sugars.

In raffinose W2b is not observed at any S/P value in samples containing proteins: the contribution of W2a is dominant, and its population almost constant, in all protein containing samples. Taking into account that raffinose stable crystalline state is pentahydrate, an unusually large coordination number for crystallization water in sugars, it is unsurprising that this sugar may interact with more water molecules than the others. However this interacting water is likely not strongly coordinated to the sugar, as it contributes mostly to an indistinct bulk-like W2 population.

**Figure 7:**
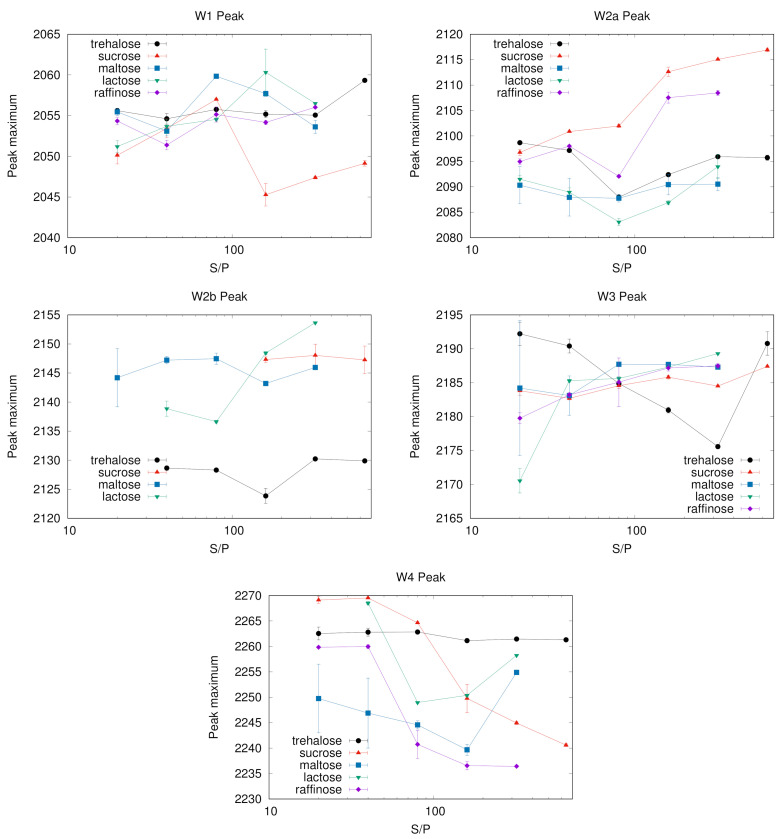
Peak frequencies of various sub-bands. As in the previous figures, the vertical bars are standard deviation on the population. The instrumental resolution uncertainty, estimated around 0.3 cm${}^{-1}$, is not included in the evaluation of the bars for sake of clarity. In almost all cases, within the errors, the populations interconvert with the sugar:protein ratio with no peak shift; the remarkable blue and red shifts occurring for W2a and W4 in sucrose, and for W3 in trehalose, could be attributed to the peculiarity of trehalose and sucrose in modulating the strength of the protein-water-sugar interactions established in the samples.

Figure 7 reports the maximum peak frequency of W1–W4 sub-bands as a function of S/P ratio. Changes in the maximum peak frequency with the system composition are evident in some matrices. This is the case of sucrose and raffinose for the sub-bands W2a and W4, which have a clear trend. In some other cases, as for W2b in all saccharides but lactose or W3 for all but trehalose, the peak frequency is roughly constant within the errors. Others components have instead a less clear behaviour, as e.g., W1 that for sucrose shows a “jump” corresponding to the change in band shape (see Figure 2, left panel).

With the only exception of the W3 sub-band, results point out lower changes in trehalose compared to the other saccharides, in particular for the chaotropic W1 and ice-like W4 bands. The case of the W3 component in trehalose deserves some further comment. Its population increases with the sugar concentration up to S/P = 320, then it decreases in correspondence of S/P = 640, recovering the population reached at the S/P = 320 value in samples without protein (see in Figure 1). The peak frequency exhibits the opposite behaviour with S/P: we observe a red shift with S/P up to S/P = 320; by adding more sugar, up to S/P = 640, the peak frequency shift reverts.

As W3 arises from a kosmotropic environment of water molecules, we already noticed that its population increases with increasing S/P in all systems, making this sub-band likely to correspond to water not directly involved with the embedded protein. Taking into account the behaviour of W3 peak frequency we suggest to attribute it to water mostly in contact with trehalose molecules which are not protein-bound, but either in mainly trehalose domains or involved in irregular sugar patches formed around the proteins, which set up at very high sugar concentration [22]. These patches would provide a potential kosmotropic environment for water though less rigid than in water-sugar only structures. The more sugar is added, the larger is the size of these irregular patches, and the higher is the population of water involved in them, which contributes to the observed red-shift with S/P. This hypothesis would agree also with the observation of the blue shift from S/P = 320 to the highest sugar concentration investigated (S/P = 640). Indeed, in this extreme condition the patches would be large enough to approach the structure of the trehalose-water domains, and a larger fraction of the sample would become more similar to a pure binary sample in which stronger water-trehalose interactions are dominant.

To summarize, some sugars (trehalose, maltose and lactose in our cases) are able to provide protein-water-sugar structures where different families of water molecules mostly just “interconvert” by changing the system’s composition, leaving almost unaltered the average oscillator strength of each sub-population. In other sugars, as sucrose or raffinose, depending on the protein or sugar content, looser or more rigid HB networks set up in the same class of water, leading to red or blue shift of the corresponding sub-band.

To better clarify this aspect, we try to relate the IR data here reported to some previous structural data, namely SAXS results on trehalose, sucrose, lactose and maltose matrices [16,51]. SAXS data showed that some inhomogenity is present in these samples. In details, two types of domains were identified, a protein-containing background, which could be assumed as a more chaotropic environment for water, and protein depleted isles, composed mostly by sugar and water, where water molecules sense a more kosmotropic enviroment. In the different sugars, these inhomogeneities emerge with different average sizes, and likely different composition, spanning from small clusters in maltose, likely mainly constituted by sugar [52,53] to large domains in sucrose not very different from the background, as shown by their low contrast.

Highly contrasted areas could be considered as distinct water reservoirs. At different S/P ratios, besides changes in number and dimension of these regions, the residual water population (both chaotropic and kosmotropic waters) might either distribute differently in one or the other domain or alter the domain structure. This reflects in the behaviour of the maximum peak frequency, presently reported. Indeed, in the case of maltose, lactose and trehalose, water population interconversion is dominant, with almost no peak frequency shift for the single sub-bands.

The different behaviour noticed in some classes and in particular for the chaotropic W1, bulk-like W2a and the kosmotropic W4 peaks of sucrose suggests instead that difference in water content reflects in alteration of the domain structure. This supports the molecular hypothesis we proposed on the basis of SAXS results: in this system the domains are scarcely different from the background and the effect of altering the S/P ratio is more likely a widespread modification of both domain and background, with the effect of altering the WAB sub-bands peak frequencies, than a different partition of the components between the two well defined domains.

As for the W2a population in sucrose, it increases at low S/P while the peak frequency decreases. This class of water molecules could be attributed to water more interacting with the protein than with sucrose molecules, and therefore sensing a more chaotropic environment in sugar-poor systems.

The remarkable red shift observed in the W4 band in sucrose by increasing the S/P ratio is in agreement with results from EPR measurements of a nitroxide probe in sugar matrices of a membrane protein [36], at different sugar/protein ratio. According to the reported results, at low protein concentration (high S/P values), the matrix appears heterogeneous, including sucrose polycrystalline clusters, some amorphous regions, and regions where the protein, the nitroxide probe and the residual water retain large mobility. Increasing the protein concentration (low S/P values) induces the formation of HBs between the sucrose matrix and protein surface. This, beside impairing the formation of sucrose clusters, promotes the set up of a progressively more extended HB network, which involves the embedded protein, the nitroxide probe, sucrose and residual water molecules. This behaviour of the W4 peak frequency at low S/P is observed also in other sugar systems, with the exception of trehalose where a similar behavior occurs for the W3 sub-band. Overall, for the “kosmotropic” classes of water (W3 and W4), a blue shift is observed by increasing the protein concentration. The existence of protein–residual-water–sugar HBs structures as the ones invoked in Malferrari et al. [36] was actually reported time ago, on the basis of results from MD simulations of MbCO embedded in trehalose or sucrose or maltose systems [16,20]. It was showed that a substantial fraction of residual water in the system bridges protein residues and sugar molecules, and that this water is engaged in multiple HBs with both the components. Similar results were also reported in a simulative study of single and multi-protein systems embedded in trehalose [28,49,54,55].

## 3. Materials and Methods

For sample preparation lyophilized ferric horse myoglobin (with purity >99.4%), maltose and other reagents (all from Sigma, St. Louis, MO, USA) and sucrose (Fluka GmbH, Buchs, Switzerland) have been used without preliminary purification, while trehalose (Hayashibara Shoji Inc., Okayama, Japan) and lactose (Galeno srl, Comeana, Italy) have been utilized after recrystallisation from aqueous solution.

All the samples have been prepared from solution of identical composition, changing only the sugar. Particular care was taken because WAB shape is strongly dependent on detailed composition (sugar, buffer, co-solutes, etc.) [44]. The starting solutions have been prepared with the same molar concentration of monosaccharide units, rather than at the same nominal molar concentration, to ease the comparison among all the saccharides, since raffinose, as a trisaccharide, has a sizably different molecular weight with respect to the disaccharides. The starting solution for samples without myoglobin contained monosaccharide units 0.8 M, which corresponds to raffinose 0.27 M and disaccharides 0.4 M. The starting solution for samples with myoglobin contained different monosaccharide-units/protein ratios (S/P), in the range 20–640. With the aim at obtaining solutions with roughly the same solute mass content, both protein and sugar concentration have been regulated, obtaining the concentrations reported in Table 2 and Table 3. Phosphate buffer 20 mM (K${}_{2}$HPO${}_{4}$ + KH${}_{2}$PO${}_{4}$, pH 7) has been added to all the starting solutions to avoid possible Mb unfolding due to pH imbalance; its presence is not necessary in solutions without Mb, but it has been added for consistency. Since the starting solutions have been prepared with Mb in oxidized form (*met*-myoglobin), they have been pre-equilibrated with gaseous CO, adding sodium dithionite (∼5 mM) to reduce Fe(III) to Fe(II).

The amorphous samples have been obtained directly on the measurement support (CaF${}_{2}$ round windows with a diameter of ∼13 mm), by layering ∼150 μL of the starting solutions on the window surface, obtaining a film. Subsequently, the samples on their supports have been first desiccated at room temperature in the presence of silica gel and then dried for ∼20 h under a low pressure CO atmosphere. After this drying stage another CaF${}_{2}$ window has been put on top of each sample, in order to clamp the film between the two windows and avoid any possible loss of material, before a second drying stage under vacuum. After this stage the samples were ready for measurements. Actually each measurements was preceded by a period of water content monitoring at 300 K, by observing the $\nu_{2}+\nu_{3}$ water combination band at 5200 cm${}^{-1}$ up to its stabilization, to allow the loss of possible residual water adsorbed on the windows out of the sample [41].

We studied only highly desiccated samples. Provided that the system is homogeneous, saccharides are more effective at low water contents, where direct protein-sugar interactions suppress the dynamics. In these conditions the peculiarities of each saccharide are more evident [8,16].

The whole strong dehydration protocol above described has been designed to achieve this highly dehydrated state. It is important to highlight that it is actually possible to extract more water from these samples, by e.g., increasing the temperature or by allowing the sample to crack and turn to dust. However the systematic protocol reported above allowed us to obtain reproducible samples characterized by: (a) very low hydration, (b) sample macroscopic homogeneity and (c) amorphous state without crystallization. The downside of this procedure is that the resulting samples might have different water content after desiccation, because of the different saccharide and protein hygroscopicity. Samples with high sugar concentration would be naturally able to retain a larger water content, exactly as samples prepared from more hygroscopic sugars. With our protocol the final water content is a stationary value reached by the specific system at “equilibrium”. We want to stress that drifting apart from these equilibrium values, e.g., by forcing a fixed final water content irrespective of the sugar and protein content, would likely give unstable systems as a result, with extensive risks of sample crystallization or other form of phase separation. These are both phenomena detrimental to the matrix preservation properties [56], which can be recognized by peak duplication and enhanced opacity (low baseline transmittance) [7,32,57].

The spectra have been recorded with 1 cm${}^{-1}$ resolution with a Jasco FTIR-410 spectrophotometer connected to a constant flux of dry nitrogen. Each measurement is the result of an average of 250 scans at 300 K, to improve the signal/noise ratio. Three to eight samples have been measured for each system. In the Figure 1, Figure 2, Figure 3, Figure 4 and Figure 5, we report a representative spectrum chosen among those measured, while the populations and peak maximum frequencies, reported in the tables and figures, are obtained by averaging all the measurements for each sample.

### The Water Association Band

The WAB is an infrared band, which can be observed in the range 2000–2500 cm${}^{-1}$ in water and hydrated systems. Its presence is usually well evident in that wavenumber range, as there are few interfering bands [58]. It is relatively weak and with a broad shape, as expected for a combination band. This band has a still unclear origin, and a few hypotheses have been proposed from a simple second overtone of the water librational mode $\nu_{L2}$, which correctly describes WAB beaviour in ice, but not in liquid water, to a combination of bending and librational fundamental modes, which would explain the occurrence of WAB only in condensed systems and correctly describe it in liquid water, but fails in ice [59]. However, both these hypotheses can be accepted with some anharmonic corrections or modifications for intermolecular contributions [58,60], which involve translational modes in liquids [61]. High-level quantum mechanical calculations have been used more recently to try to solve the WAB issue, but an accurate description of the broadening has not been obtained yet, because of the inability to correctly describe the nuclear quantum dynamics [62,63]. The attributions based on intermolecular modes can explain the area reduction and the peak blue shift with decreasing temperature and in structured environments. Indeed, the wave function superposition with other hydrogen-bonded molecules decreases with increasing temperature, due to the HB network relaxation and to the increased librational flexibility. This makes to decrease the band frequency and intensity [59,64]. WAB high and low frequency components could respectively be attributed to water molecules that interacts through strong and weak HBs [64].

This high dependence on the environment makes the WAB an useful marker for hydrogen bonded molecules (osmolytes, denaturants, salt) interacting with water, as well as a probe for crowding and confinement [44,64]. In saccharide matrices, where strong water-sugar interactions occur, a strong dependence of WAB shape and peak frequency on sugar and hydration has been reported [41,44].

WAB has a smooth Gaussian shape in liquid water and approximately also beyond 30% hydration, whereas it becomes structured by lowering the water content [33,41,42,44]. An extensive study of WAB in trehalose and trehalose-myoglobin matrices is reported in Giuffrida et al. [44]; in this study different water subpopulations were identified, and assigned to the various sub-bands that can be used to decompose the WAB. This was done by analyzing the effect of Hofmeister salts on the band, exploiting their well known ability to modify the local order, altering the HB network strength. By connecting the known salt effect to the signal modifications, one could identify probes for weak or strong HBs [65]. Results allowed to ascribe the sub-bands to water in different chaotropic/kosmotropic environments, as well as to differentiate bulk-like and ice-like water. As done in Giuffrida et al. [44], in this work the band was normalized and analysed by making use of the lower possible number of Gaussian curves to define the band profile. We decided to employ Gaussian curves because of the high level of inhomogeneity expected, as each sub-band would not be related to a distinct structure, but to a more blurred family of HB water networks that can exchange water with one another. From the original spectra a background curve was subtracted before the analysis. This background curve was always a constant plus a Gaussian tail, introduced to describe that part of the very strong CH stretching bands (2800–3000 cm${}^{-1}$) falling within the spectral range of the WAB. The resulting curves were normalized, because WAB area has only a rough proportionality to water content, at variance with the $\nu_{2}+\nu_{3}$ water combination band, and our object of study is the relative fraction of sub-bands, which is put in evidence by normalization. However, when interpreting results, some points have to be taken into account: (a) WAB is to be considered more an HB marker than a water marker, but it cannot be observed in the absence of water molecules; (b) the sub-bands are wide and largely superimposed, hence large errors might occur; (c) there is no direct relationship between a sub-band and “single IR species”, the components arise from HB families and therefore (d) a given IR peak cannot be related to a specific HB pair [66].

**Figure 8:**
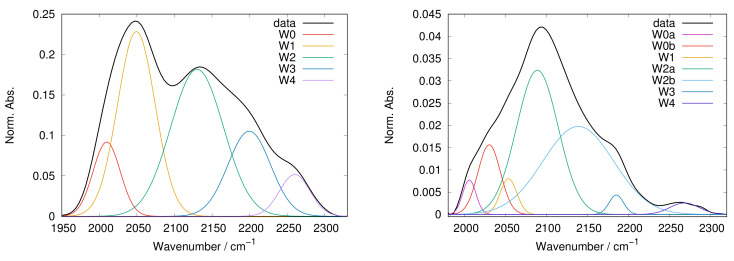
Representative WAB fitting in terms of five components (**left panel**), a sample of trehalose:NaClO${}_{4}$ 2:1, reported from [44]), and seven components (**right panel**), a sample of myoglobin-lactose at S/P = 40). In both figures, the background has been subtracted, but the band is not normalized.

With respect to the analysis performed to characterize the WAB with water-salts-trehalose samples [44], which showed that a decomposition in terms of five recurring sub-bands fairly described the band, in this case seven sub-bands were needed. The two new components arise from the splitting in two of the previous W0 and W2 bands, and have been named W0a, W0b and W2a, W2b for sake of consistency with the naming scheme we already used in [44]. Figure 8 shows two examples of analysis in terms of five and seven bands (left and right panels). The frequency range for each sub-band (Wn, with n from 0 to 4), alongside the attribution proposed, is reported in Table 4.

Five sub-bands (W0b, W1, W2a, W3 and W4) were found in all samples, with the other two (W0a and W2b) needed only in some systems; note that this means that sub-band duplication is not a general need for the data analysis. One could wonder at the width of their frequency ranges, which are large for normal IR bands in mostly homogeneous systems, as the present ones. But this could have been expected, since also in pure water the WAB is extremely broad, with 300 cm${}^{-1}$ width (full width at half height).

W0 and W1 have been attributed to water molecules in non-structured environments, as in the presence of structure breakers. In the present samples W0a,b are observed mainly at high protein content, hence we may suppose that they are related with water interacting with protein surfaces. Beside a general reduction with increasing sugar content, W0a,b bands do not show neither significant peak shifts nor population interconversion with other bands, and their behavior is not even significantly different among the different sugars, hence we will not deepen the analysis of them.

The W1 component can be ascribed to water embedded in a chaotropic environment, as a substantial population was found in the presence of neutral to chaotropic anions [44]. As above noticed W1 increases with increasing sugar content, hence we suggest to attribute it to water in a chaotropic environment, but mostly in the sugar domain. W1 is also the first component that appears when dehydrating a protein-saccharide amorphous system, along with the bulk-like component W2 [41,42].

W2 likely arises from water molecules whose environment is similar to liquid water (bulk-like water), not necessarily water interacting with water, but also water molecules involved in HBs patterns as in bulk water. This band is clearly present and usually the most populated in pure water, and the first to be populated upon hydration. On dehydrating saccharide and saccharide-protein systems, one observes a progressive narrowing and depopulation of the W2 band, then the development of the other components. It has been also observed that W2 peak maximum might shift following dehydration, this in dependence of the matrix composition, because of the different interactions taking place and of their alterations with decreasing water content [42]. In the present samples W2 is splitted in two components, likely representing weakly destructured or weakly structured water, with W2a always present and usually dominant, while W2b appearing and increasing with increasing S/P ratio. The large population of these sub-bands in all the samples indicates that most of water undergoes only a weak strain, laying in slightly destructured or slightly structured environments, both scarcely dissimilar from that of liquid water.

W3 and W4 both correspond to more structured water. In the present samples both W3 and W4 increase with increasing S/P, as expected as sugars are more kosmotropic than myoglobin. Attribution of W4 is straightforward: this sub-band can be observed on the blue side of the WAB, where the maximum of cubic ice (2255 cm${}^{-1}$) falls. In trehalose matrices an increase of W4, along with the entire high frequency side, is clearly evident upon crystallization [44]. W4 is therefore assigned to ice-like water; exactly like bulk-like water, it does not necessarily arise from crystalline ice of some form, but corresponds to water in an highly ordered environment. As crystallisation increases also W3 component, one can suggest that also this sub-band arise from water in ordered environments. In spite of this, W3 population generally reduces upon hydration and it is not perceivable in pure water, so it is likely related with water interacting with solutes, and not from structures that form in pure water.

In this respect, the analysis of pure sugar-water matrices shows that the W3 component is present in almost all sugar samples, while it is a minoritary band in sucrose, where it is dwarfed by the bulk-like W2b component. We attribute this finding to the peculiar sugar-water interaction. Indeed, it is well known that at low water content sucrose could form intramolecular HBs [40,52], leaving fewer available sites able to interact with water.

## 4. Conclusions

Water plays a fundamental role in the modulation the matrix structure and dynamics and the protein-matrix coupling in amorphous saccharide matrices at low hydration. In the present work, we investigate matrices containing different sugars and different sugar/protein ratios by exploiting the Water Association Band (WAB), which has been proven to be a valuable probe for the behavior of water molecules as well as for the HB networks in which they take part.

Five to seven sub-bands can be identified in almost all samples, whose population and characteristics change with sugar/protein ratio, from protein-rich to sugar-rich samples. A differential analysis of the fractional populations and peak frequencies of the various WAB sub-bands can unravel the water behaviour and properties in such saccharide amorphous matrices. A comparison with pure water-sugar systems without proteins is carried out, providing a reference case against which to gauge the protein effects.

The novelty of this work is to show how these properties are modulated by protein and co-solute insertion in a way that is peculiar to each sugar, providing results that are in agreement with and complement the ones from more “structural” studies, both at the atomistic (simulations) and the supramolecular level (SAXS). In particular, the main findings could be summarized as follows:

(i) for some sugars as trehalose or maltose, HB networks involving sugar, water and proteins residues can set up in the samples, where residual water smoothly switches (“interconverts”) from the protein to the sugar domain, keeping however unaltered the strength of the interactions both with the protein and the sugar.
(ii) for some other sugars, and in particular for sucrose, looser or more rigid HB networks set up in the same class of water. Both the blue shift of bulk-like water and the red shift of kosmotropic water observed by increasing the sucrose content, are indicative of the set up of water domains “separated” from sugar and protein domains, as already hypothesized on the basis of EPR results in sugar matrices [36].
(iii) in comparison with the other saccharides, in trehalose the composition changes lead to a more gradual rearrangement of the local structures, shaped by protein, water and sugar in the sample, as evidenced by smooth variation of almost all the WAB fractional populations and relative peak frequencies with S/P.

This indicates that a trehalose matrix is prone to accommodate smoothly variations in water and protein content and is therefore able to provide a friendly host environment to the embedded protein in a wide range of sugar, protein, and water concentrations.

We believe that results from the present work have strong implications in the context of biopreservation by saccharides, providing a guidance to the proper formulation for long term storage of biomolecules at very low hydration. In this respect, the trehalose peculiarity is one more time highlighted.

## Figures and Tables

**Table 1:** Water/Saccharide molar ratio (W/S). Data are taken from [37].

S/P	Trehalose	Sucrose	Maltose	Lactose	Raffinose
20	3.8	0.8	1.2	2	1.2
40	1.8	0.4	0.5	0.4	1.0
80	0.3	0.3	0.3	0.2	0.3
160	1.4	2.2	1.2	2.1	2.1
320	2	3.3	2.4	3.2	5.6
640	2.5	1.1	-	-	-
*∞*	3.0	0.8	4	5.3	6.8

**Table 2:** Molar concentration of disaccharides and protein used to prepare the samples.

S/P	Disaccharide (mM)	Mb (mM)
20	80	8
40	140	7
80	200	5
160	240	3
320	320	2
640 ${}^{o}$	320	1
*∞*	400	–

^o^ Samples at this ratio were prepared only for sucrose and trehalose.

**Table 3:** Molar concentration of protein and raffinose used to prepare the samples.

S/P	Raffinose (mM)	Mb (mM)
20	50	8
40	90	7
80	130	5
160	160	3
320	210	2
*∞*	270	–

**Table 4:** Frequency intervals and attributions for WAB sub-bands and duplications

Name	Frequency Range	Attribution
W0a	2000–2010 cm${}^{-1}$	Strongly destructured
W0b	2015–2035 cm${}^{-1}$	Strongly destructured
W1	2045–2065 cm${}^{-1}$	Destructured
W2a	2080–2120 cm${}^{-1}$	Weakly destructured (*Bulk like*)
W2b	2120–2160 cm${}^{-1}$	Weakly structured (*Bulk like*)
W3	2170–2200 cm${}^{-1}$	Structured
W4	2230–2270 cm${}^{-1}$	Strongly structured (*Ice like*)

## Data Availability

The results (outcome of the data analysis) presented in this study are available within this article. The original data, which are in common with Giuffrida et al. [37], are available at request from the authors.

## Abbreviations

The following abbreviations are used in this manuscript:

WAB	Water Association Band
COB	Carbon Monoxide Band
MbCO	Carbonmonoxy Myoglobin
FTIR	Fourier Tansform Infrared
HB	Hydrogen Bond
